# Evaluation of lipid ratios and triglyceride-glucose index as risk markers of insulin resistance in Iranian polycystic ovary syndrome women

**DOI:** 10.1186/s12944-020-01410-8

**Published:** 2020-11-08

**Authors:** Asma Kheirollahi, Maryam Teimouri, Mehrdad Karimi, Akram Vatannejad, Nariman Moradi, Nasrin Borumandnia, Asie Sadeghi

**Affiliations:** 1 Department of Comparative Biosciences, Faculty of Veterinary Medicine, University of Tehran, Tehran, Iran; 2 Department of biochemistry, School of Paramedicine, Shahroud University of Medical Sciences, Shahroud, Iran; 3 Department of Epidemiology and Biostatistics, School of Public Health, Tehran University of Medical Sciences, Tehran, Iran; 4 Cellular and Molecular Research Center, Research Institute for Health Development, Kurdistan University of Medical Sciences, Sanandaj, Iran; 5 Urology and Nephrology Research Centre, Shahid Beheshti University of Medical Sciences, Tehran, Iran; 6 Student Research Committee, School of Medicine, Kerman University of Medical Sciences, Kerman, Iran; 7 Department of Clinical Biochemistry, Faculty of Medicine, Kerman University of Medical Sciences, Kerman, Iran

**Keywords:** Polycystic ovary syndrome, Insulin resistance, Fasting triglyceride-glucose index, Triglyceride to high-density lipoprotein cholesterol, Total cholesterol to high-density lipoprotein cholesterol, Homeostasis model assessment for insulin resistance, Dyslipidemia

## Abstract

**Background:**

Insulin resistance has a vital role in the pathophysiology of polycystic ovary syndrome (PCOS). Previous investigations have shown that some lipid ratios could be a simple clinical indicator of insulin resistance (IR) in some disorders and ethnicities. The present study was conducted to evaluate the correlation between triglyceride to HDL-cholesterol (TG/HDL-C), total cholesterol to HDL-cholesterol (TC/HDL-C), as well as fasting triglyceride-glucose (TyG) indices with IR (as measured by homeostasis model assessment of IR (HOMA-IR), quantitative insulin sensitivity check index (QUICKI) and fasting glucose to insulin ratio (FGIR)) among the Iranian women diagnosed with PCOS.

**Methods:**

In the current study, a total of 305 women with PCOS were evaluated. TG/HDL-C, TC/HDL-C, and TyG indices were calculated. Fasting insulin level was measured using ELISA technique. IR was defined as a HOMA-IR value of ≥2.63, FG-IR value of < 8.25, and QUICKI value of < 0.33.

**Results:**

The insulin-resistant (IR) and insulin-sensitive (IS) groups, established by the HOMA-IR, FG-IR, and QUICKI values were different in terms of TG/HDL-C, TC/HDL-C, and TyG indices. These indices were associated with IR even after adjusting for age and BMI. ROC curve analyses showed that TyG, TG/HDL-C, and TC/HDL-C strongly predicted HOMA-IR with area under the curve (AUC) of 0.639, 0.619, and 0.623, respectively (*P* < 0.05). Further, TC/HDL-C was a good predictor of FG-IR with AUC of 0.614 (*P* = 0.04).

**Conclusion:**

TyG, TG/HDL-C, and TC/HDL-C indices might be good indicators of IR among Iranian women diagnosed with PCOS.

**Supplementary Information:**

**Supplementary information** accompanies this paper at 10.1186/s12944-020-01410-8.

## Introduction

Polycystic ovary syndrome (PCOS) is a complex hormonal disorder commonly occurring in women of reproductive age. It is characterized by ovulatory dysfunction, hyperandrogenism, and polycystic ovarian morphology (PCOM) under B-ultrasound imaging [1]. The prevalence of PCOS is approximately 5–15%. It is the most common cause of infertility in women [1]. PCOS shares many features with metabolic syndrome where the majority of women diagnosed with PCOS (44–85%) exhibit insulin resistance (IR) and compensatory hyperinsulinemia, regardless of body mass index (BMI) [2–4]. An increase in insulin and androgen levels interrupts the follicular growth, which in turn leads to the irregular menstrual cycle, anovulatory subfertility, and accumulation of immature follicles [2, 5]. Although, the pathogenesis of PCOS is not fully elucidated yet, a growing body of evidence shows that IR has a central role in the pathophysiology of PCOS and is associated with increased risk of metabolic disorders including type 2 diabetes mellitus (T2DM), dyslipidemia, nonalcoholic fatty liver disease (NAFLD), and cardiovascular disease (CVD) [6]. It has been demonstrated that modulating of IR results in a significant improvement in PCOS complications [7, 8]. Therefore, it is of paramount importance to evaluate IR among this high-risk population.

The hyperinsulinemic-euglycemic glucose clamp technique is the gold standard for the diagnosis of IR [9]. This technique is, however, time-consuming, labor-intensive, costly and technically challenging [10], and for this reason, surrogate markers including homeostasis model assessment for insulin resistance (HOMA-IR), the quantitative insulin sensitivity check index (QUICKI), and the fasting glucose to insulin ratio (FG-IR) have emerged to estimate IR [11]. The clinical efficacy of these markers is limited because of the absence of standardization, high cost and the lack of availability of the insulin assay technique [12]. Hence a simple and more available marker for predicting IR can be valuable and cost-effective in routine clinical practice [12]. Previous studies have suggested that the fasting triglyceride-glucose (TyG) index and the triglyceride to high-density lipoprotein cholesterol (TG/HDL-C) ratio closely associate with IR [13–15]. Moreover, some studies have shown that total cholesterol to high-density lipoprotein cholesterol (TC/ HDL-C) ratio correlates with IR and CVD risk [15]. It is worth mentioning that the association between lipid ratios and IR may differ according to ethnicity and some indices may not be applicable to predict IR in particular populations [16–18]. Few studies have been carried out on the association between IR and lipid ratios among Iranian women with PCOS.

The current study was conducted (i) to investigate the association between TG/HDL-C, TC/HDL-C, and TyG indices with IR (as measured by HOMA-IR, QUICKI, and FG-IR indices); (ii) to determine the cut-off points of the TG/HDL-C, TC/HDL-C, and TyG indices for predicting IR, and (iii) to evaluate the diagnostic utility of these markers in detecting IR among the Iranian women diagnosed with PCOS.

## Materials and methods

### Study participants

A total of 305 women with PCOS were recruited from Shahid Bahonar Hospital, Kerman, Iran, from May 2018 to January 2019. The sample size was calculated using the appropriate formula proposed in an article by Hajian-Tilaki [19]. The age of the participants ranged from 20 to 40 years old, with a BMI of 17–35 kg/m^2^. The case group included subjects newly diagnosed with PCOS, based on the 2003 Rotterdam Criteria, which dictates the following criteria: PCOM on ultrasound imaging, hyperandrogenism, and/or oligo- or anovulation [20]. Following diagnosis by a gynecologist, patients completed a questionnaire about the demographic characteristics, history of the disease, and use of medications. Exclusion criteria comprised patients taking anti-lipidemic drugs, anti-hypertensive, weight-loss and hormonal drugs, those suffering from hypertension, endocrine disorders, or CVD, and pregnant women. The present research was approved by the Ethics Committee of Kerman University of Medical Sciences (IR. KUMS.REC.1399.208), and was carried out according to the declaration of Helsinki. An informed consent was obtained from all the subjects before enrollment in the study.

### Anthropometric and laboratory measurements

BMI was calculated using standard formula [body weight (kg)/height squared (m^2^)]. Blood samples were taken from the subjects after an eight-hour fasting period at the follicular phase of their menstrual cycle. Biochemical parameters including fasting blood glucose (FBG), TC, TG, LDL-C, and HDL-C were evaluated using the commercially available kits (Pars Azmoon, Tehran, Iran). The circulating levels of fasting insulin were measured using an ELISA kit (Monobind Inc., California, USA). The serum levels of follicle-stimulating hormone (FSH), luteinizing hormone (LH), free T4, and homocysteine were evaluated using the ELISA kits (Pishtaz Teb, Tehran, Iran), according to manufacturer’s instructions. Details about the inter- and intra-assay coefficients of variation (CV) for biochemical parameters have been previously reported [21].

The TG/HDL-C, TC/HDL-C, and TyG indices were determined using the following calculations respectively: TG (mg/dL)/HDL-C (mg/dL), TC (mg/dL)/HDL-C (mg/dL) and Ln [TG (mg/dL) × FBG (mg/dL)]/2.

IR was defined as a HOMA-IR value of ≥2.63, FG-IR value of < 8.25, and QUICKI value of < 0.33. The cut-off points for HOMA-IR and QUICKI were determined according to the previous studies [22, 23]. In the case of FG-IR, the cut-off value of 8.25 was obtained by ROC analysis based on the QUICKI cut-off value of 0.33. HOMA-IR was calculated as: [FBG (mg/dL)] × [fasting insulin (μU/ml)]/405 [24]. FG-IR was determined as the fasting glucose (mg/dl)/fasting insulin (μU/ml). QUICKI was calculated using the following formula: 1/(log fasting insulin [μU/ml] + log glucose [mg/dl]) [24]. Subjects were divided into insulin-resistant (IR) and insulin-sensitive (IS) groups based on HOMA-IR, FG-IR, and QUICKI indices. First, clinical and biochemical parameters were compared between the two groups. Then, the correlations of these parameters with HOMA-IR, FG-IR, and QUICKI indices were analyzed.

### Statistical analysis

Statistical analyses were performed using the IBM SPSS Statistics 16.0 software (IBM SPSS, Chicago, IL). The normal distribution of the variables was determined using the Shapiro–Wilk test. Continuous variables with normal distribution were presented as mean ± standard deviation (SD), while skewed variables were presented as median and interquartile range (IQR). Statistical difference between the IR and IS groups was assessed using the Student’s t-test and Mann–Whitney U test. Pearson’s or Spearman’s correlation coefficients was calculated for correlation analyses. Logistic regression and ROC curve analyses were used to assess the ability of lipid ratios and TyG to predict IR according to HOMA-IR and FG-IR indices in PCOS patients.

## Results

### Clinical and biochemical parameters of the study population

Table 1 shows the general characteristics, biochemical parameters, and hormonal features of the subjects in the IR and IS groups, based on HOMA-IR and FG-IR values. It is important to note that IR exhibits a direct relationship with HOMA-IR and an indirect one with FG-IR and the QUICKI indices. Due to similar results with FG-IR, comparison based on QUICKI index is not shown.

**Table 1:** Clinical and biochemical parameters of the study population according to the FG-IR and HOMA-IR indices (positive and negative)

		FG-IR			HOMA-IR		
Variables	Total PCOS	Negative	Positive	***P***-value	Negative	Positive	***P***-value
		***n*** = 275 (≥ 8.25)	***n*** = 30 (< 8.25)		***n*** = 275 (< 2.63)	***n*** = 30 (≥ 2.63)	
**Age** (years)	29.94 ± 4.56	29.82 ± 4.55	31.1 ± 4.51	0.14	29.85 ± 4.56	30.77 ± 4.52	0.29
**BMI** (kg/m^2^)	26.62 ± 4.19	26.62 ± 4.16	26.63 ± 4.51	0.98	26.66 ± 4.22	26.22 ± 4.01	0.58
**Insulin** (μU/mL)	4.34 (2.8–7.4)	4 (2.67–6.41)	13.2 (11.9–14.83)	< 0.001	4 (2.67–6.41)	13.2 (12.15–14.83)	< 0.001
**HomoCys** (μU/mL)	11.57 (8.87–15.4)	11.9 (9.07–15.4)	9.92 (7.97–14.02)	0.077	11.7 (9.19–15.4)	9.83 (7.68–13.47)	0.04
**Free-T** (pg/mL)	3.25 ± 1.15	3.25 ± 1.13	3.21 ± 1.38	0.84	3.26 ± 1.15	3.14 ± 1.2	0.59
**LH** (IU/L)	6.48 (4.25–9.45)	6.48 (4.4–9.5)	6.05 (3.46–8.6)	0.21	6.48 (4.4–9.5)	6.46 (3.44–8.63)	0.34
**FSH** (IU/L)	6 (4.44–7.55)	6 (4.47–7.54)	6.15 (4–8.79)	0.91	6 (4.47–7.5)	6.6 (4.07–8.83)	0.75
**TG** (mg/dL)	116 (86–156)	116 (86–156)	117 (81–156.75)	0.71	114 (84–155)	125 (105–205.65)	0.07
**FBG** (mg/dL)	89.57 ± 9.48	89.58 ± 9.53	89.4 ± 9.15	0.92	88.69 ± 8.87	97.6 ± 11.16	< 0.001
**TC** (mg/dL)	172.46 ± 36.24	171.56 ± 36.7	180.72 ± 31.1	0.18	171.59 ± 36.26	180.46 ± 35.69	0.20
**LDL-C** (mg/dL)	98.54 ± 29.08	98.64 ± 29.54	97.63 ± 24.86	0.85	98.12 ± 29.01	102.38 ± 29.86	0.44
**HDL-C** (mg/dL)	44 (38–49.6)	44 (38–50)	43 (36.25–46.25)	0.18	44 (39–50)	41 (34–47)	0.048
**TyG**	4.62 (4.62 ± 0.24)	4.62 ± 0.24	4.63 ± 0.23	0.73	4.61 ± 0.23	4.74 ± 0.25	0.005
**TG/HDL-C**	2.68 (1.84–3.78)	2.68 (1.84–3.72)	2.73 (1.85–4.71)	0.75	2.66 (1.81–3.69)	3.32 (2.18–5)	0.033
**TC/HDL-C**	4 ± 1.13	3.95 ± 1.12	4.44 ± 1.15	0.024	3.94 ± 1.09	4.55 ± 1.36	0.005
**FG-IR**	20.76 (11.9–20.76)	21.79 (13.78–34.46)	7.07 (5.57–7.57)	< 0.001	21.79 (13.78–34.46)	7.48 (5.57–8.46)	< 0.001
**HOMA-IR**	0.98 (0.61–1.65)	0.89 (0.56–1.43)	3.1 (2.6–3.41)	< 0.001	0.89 (0.56–1.42)	3.14 (2.93–3.5)	< 0.001
**QUICKI**	0.39 ± 0.05	0.4 ± 0.04	0.32 ± 0.01	< 0.001	0.4 ± 0.04	0.32 ± 0.01	< 0.001

The cut-off point for QIUCKI index was equal to 0.33 based on 90% percentile (positive < 0.33). Parametric data are shown as mean ± standard deviation. Non-parametric data are given as median and interquartile range [Q1-Q3]

The study population had a mean age of 29.94 ± 4.56 years, an average BMI of 26.62 ± 4.19 kg/m^2^, and a mean fasting insulin level of 5.62 ± 3.83 μU/mL.

In general, 9.83% of the subjects had a HOMA-IR value of ≥2.63, FG-IR value of < 8.25, and QUICKI value of < 0.33, including the IR group. There was no difference between the IR and the IS groups in terms of age, BMI, free T4, LH, FSH, TG, TC, and LDL-C levels (Table 1). The FG-IR- and QUICKI-positive groups had significantly higher circulating levels of fasting insulin, TC/HDL-C ratio, and HOMA-IR levels (*P* < 0.05), and significantly lower FG-IR and QUICKI values (*P* < 0.001) when compared to their negative counterparts (Table 1). In addition, the HOMA-IR-positive group exhibited significantly elevated levels of FBG, fasting insulin, TyG, TC/HDL-C, TG/HDL-C, and HOMA-IR values and significantly lower levels of homocysteine, HDL-C, FG-IR, and QUICKI values when compared to their negative counterparts.

### The correlation between the FG-IR, QUICKI, and HOMA-IR indices with clinical and biochemical parameters

Table 2 shows the correlations between FG-IR, QUICKI, and HOMA-IR indices with the clinical and biochemical parameters before and after adjusting for age and BMI. In contrast to HOMA-IR, FG-IR and QUICKI showed an inverse correlation with clinical and biochemical parameters related to IR. For instance, fasting insulin, TG, TC, TyG, TC/HDL-C, and TG/HDL-C were negatively associated with FG-IR and QUICKI indices (*P* < 0.05), and positively associated with the HOMA-IR index (*P* < 0.05), before and after adjusting for age and BMI. Circulating homocysteine levels showed an inverse correlation with the HOMA-IR index, but a direct one with FG-IR and QUICKI indices. Serum levels of HDL-C also showed a significantly negative association with the HOMA-IR index. Moreover, FBG concentration did not correlate with the FG-IR index. It, however, showed a significantly negative correlation with the QUICKI index and a positive one with the HOMA-IR index.

**Table 2:** The correlation between FG-IR, QUICKI, and HOMA-IR indices with clinical and biochemical parameters in the women with PCOS

Variables	FG-IR		QUICKI		HOMA-IR	
	Unadjusted	Adjusted	Unadjusted	Adjusted	Unadjusted	Adjusted
**Age**	0.048 (0.4)	–	0.017 (0.77)	–	0.04 (0.48)	–
**BMI**	− 0.03 (0.6)	–	− 0.06 (0.28)	–	0.04 (0.44)	–
**Insulin**	− 0.762 (< 0.001)	− 0.765 (< 0.001)	− 0.871 (< 0.001)	− 0.872 (< 0.001)	0.983 (< 0.001)	0.98 (< 0.001)
**HomoCys**	0.168 (0.003)	0.172 (0.003)	0.128 (0.025)	0.134 (0.019)	− 0.145 (0.01)	− 0.149 (0.009)
**Free-T**	− 0.04 (0.4)	− 0.038 (0.5)	− 0.013 (0.81)	−0.011 (0.84)	− 0.026 (0.65)	− 0.025 (0.66)
**LH**	0.01 (0.86)	0.01 (0.8)	0.035 (0.54)	0.03 (0.6)	− 0.068 (0.23)	−0.061 (0.28)
**FSH**	0.04 (0.41)	0.04 (0.43)	0.037 (0.52)	0.036 (0.53)	−0.004 (0.94)	−0.005 (0.93)
**TG**	−0.143 (0.013)	−0.144 (0.012)	− 0.163 (0.004)	−0.16 (0.005)	0.17 (0.003)	0.165 (0.004)
**FBG**	−0.039 (0.5)	−0.036 (0.5)	− 0.33 (< 0.001)	−0.327 (< 0.001)	0.331 (< 0.001)	0.329 (< 0.001)
**TC**	−0.164 (0.004)	− 0.165 (0.004)	− 0.173 (0.002)	−0.172 (0.003)	0.174 (0.002)	0.171 0.003)
**LDL-C**	−0.06 (0.26)	−0.069 (0.23)	− 0.07 (0.22)	−0.069 (0.23)	0.063 (0.27)	0.057 (0.32)
**HDL-C**	0.08 (0.14)	0.079 (0.17)	0.1 (0.06)	0.099 (0.08)	−0.119 (0.038)	−0.116 (0.044)
**TyG**	−0.163 (0.004)	−0.165 (0.004)	− 0.241 (< 0.001)	−0.238 (< 0.001)	0.233 (< 0.001)	0.227 (< 0.001)
**TG/HDL-C**	−0.15 (0.009)	− 0.151 (0.009)	−0.187 (0.001)	− 0.183 (0.001)	0.214 (< 0.001)	0.209 (< 0.001)
**TC/HDL-C**	−0.175 (0.002)	− 0.175 (0.002)	− 0.207 (< 0.001)	−0.202 (< 0.001)	0.249 (< 0.001)	0.245 (< 0.001)

The data are given in the form of correlation coefficient at the significance level (*P*-value)

### Association between the lipid profile, lipid ratios, and TyG index with IR

Table 3 shows two models of the association between FG-IR and HOMA-IR indices with lipid profiles, TyG, TC/HDL-C, and TG/ HDL-C through multiple logistic regression analyses. Since the results obtained based on the QUICKI index were similar to those obtained based on the FG-IR index, they are not presented here to avoid redundancy.

**Table 3:** Association between FG-IR and HOMA-IR indices with the lipid profiles, TyG, TC/HDL-C, and TG/HDL-C indices in the women with PCOS

	Variables	Beta (SE)	FG-IR		Beta (SE)	HOMA-IR	
			OR (95% CI)	***P***-value		OR (95% CI)	***P***-value
**Unadjusted models**	**HomoCys**	−0.077 (0.043)	0.926 (0.851–1.008)	0.075	−0.098 (0.045)	0.907 (0.831–0.991)	0.03
	**TG**	0.001 (0.003)	1.001 (0.995–1.008)	0.7	0.006 (0.003)	1.006 (1–1.011)	0.062
	**TC**	0.007 (0.005)	1.007 (0.997–1.017)	0.189	0.007 (0.005)	1.007 (0.996–1.017)	0.2
	**LDL-C**	−0.001 (0.007)	0.999 (0.986–1.012)	0.85	0.005 (0.006)	1.005 (0.992–1.018)	0.44
	**HDL-C**	−0.038 (0.023)	0.963 (0.92–1.008)	0.1	−0.047 (0.024)	0.954 (0.91–1.001)	0.054
	**TyG**	0.281 (0.816)	1.325 (0.268–6.553)	0.73	2.383 (0.858)	10.832 (2.015–58.236)	0.005
	**TG/HDL-C**	0.110 (0.107)	1.117 (0.905–1.377)	0.3	0.259 (0.100)	1.295 (1.065–1.575)	0.009
	**TC/HDL-C**	0.358 (0.160)	1.43 (1.044–1.958)	0.026	0.439 (0.160)	1.551 (1.134–2.123)	0.006
**Adjusted models for age and BMI**	**HomoCys**	−0.074 (0.4)	0.928 (0.855–1.008)	0.078	−0.096 (0.045)	0.909 (0.832–0.992)	0.033
	**TG**	0.001 (0.003)	1.001 (0.995–1.007)	0.77	0.006 (0.003)	1.006 (1–1.012)	0.061
	**TC**	0.007 (0.005)	1.007 (0.996–1.017)	0.19	0.007 (0.005)	1.007 (0.996–1.017)	0.2
	**LDL-C**	−0.002 (0.007)	0.998 (0.984–1.011)	0.71	0.005 (0.007)	1.005 (0.992–1.018)	0.49
	**HDL-C**	−0.040 (0.023)	0.961 (0.919–1.006)	0.086	−0.049 (0.024)	0.952 (0.908–0.998)	0.04
	**TyG**	0.203 (0.835)	1.225 (0.239–6.288)	0.8	2.453 (0.871)	11.621 (2.109–64.03)	0.005
	**TG/HDL-C**	0.103 (0.108)	1.109 (0.897–1.37)	0.33	0.266 (0.101)	1.304 (1.07–1.59)	0.009
	**TC/HDL-C**	0.357 (0.162)	1.429 (1.04–1.961)	0.027	0.454 (0.162)	1.574 (1.145–2.165)	0.005

Serum homocysteine (0.907, 95% CI [0.831–0.991]), TG/HDL-C (1.295, 95% CI [1.065–1.575]), TC/HDL-C (1.551, 95% CI [1.134–2.123]), and TyG (10.832, 95% CI [2.015–58.236]) were associated with the HOMA-IR index; TC/HDL-C (1.43, 95% CI [1.044–1.958]) however, was associated with the FG-IR index (Model 1). Importantly, the associations remained significant after adjusting for age and BMI (Model 2). As such, serum homocysteine (0.902, 95% CI [0.832–0.992]), TG/HDL-C (1.304, 95% CI [1.07–1.59]), TC/HDL-C (1.57, 95% CI [1.145–2.165]), and TyG (11.622, 95% CI [2.109–64.03]) were associated with the HOMA-IR index, while TC/HDL-C (1.429, 95% CI [1.04–1.961]) was associated with the FG-IR index.

### ROC analysis

**Fig. 1:**
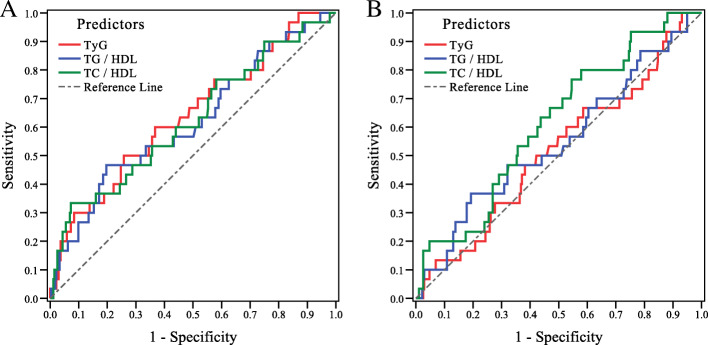
The results of ROC curve analysis regarding the predictability of TyG, TC/HDL-C, and TG/HDL-C indices in classifying the IR considering (**a**) HOMA-IR and (**b**) FG-IR in the patients with PCOS

Figure 1 shows the ROC curve for TyG, TG/HDL-C, and TC/HDL-C indices as predictors for HOMA-IR and FG-IR. TyG, TG/HDL-C, and TC/HDL-C strongly predicted HOMA-IR with AUC values of 0.639, 0.619, and 0.623, respectively (*P* < 0.05) **(**Table 4**)**. Only TC/HDL-C significantly predicted FG-IR with an AUC of 0.614 (*P* = 0.04) and the lipid profile (TG, TC, LDL-C, and HDL-C) failed to predict both HOMA-IR and FG-IR indices (Supplementary Fig. 1).

**Table 4:** The areas under ROC curve (AUC), sensitivity, specificity by the optimized cut-off points for TyG, TC/HDL-C, and TG/HDL-C indices in predicting the HOMA-IR and FG-IR indices

	Predictors	AUC (95% CI)	***P***-value	Cut-off value	Sensitivity	Specificity
**HOMA-IR**	**TyG**	0.64 (0.53–0.75)	0.012	≥ 4.65	0.63	0.60
	**TG to HDL**	0.62 (0.51–0.73)	0.033	≥ 2.80	0.60	0.56
	**TC to HDL**	0.62 (0.51–0.74)	0.027	≥ 4.00	0.60	0.57
**FG-IR**	**TyG**	0.52 (0.41–0.63)	0.76	≥4.617	0.57	0.51
	**TG to HDL**	0.55 (0.43–0.66)	0.38	≥2.687	0.50	0.51
	**TC to HDL**	0.61 (0.51–0.71)	0.041	≥ 4.10	0.60	0.58

## Discussion

IR plays a central role in the pathogenesis and complications of PCOS [25], with compensatory hyperinsulinemia being a critical factor in the progression of hyperandrogenism and reproductive disorders. As such, IR may be a preliminary risk factor for PCOS, rendering it pivotal in the management of this disease [26]. Direct and indirect methods (HOMA-IR, QUICKI, and FG-IR) for IR assessment are often complicated, expensive, and inappropriate for epidemiological studies [25]. Hence it is necessary to develop an easy-to-measure, more reasonable, and cost-effective IR assessment method for a more accurate and personalized diagnosis, treatment, and prognosis of PCOS [25].

Dyslipidemia is a common metabolic complication affecting up to 70 % of females diagnosed with PCOS [27]. Many factors are known to involve in disrupted lipid metabolism and dyslipidemia. IR plays a pivotal role mainly by stimulating lipolysis and altering the expression of lipoprotein lipase and hepatic lipase [27]. In this regard, many women with PCOS exhibit features consistent with metabolic syndrome including elevated levels of triglycerides, TC, and LDL-C, and depressed levels of HDL-C [28]. Recently, some studies have proposed that lipid ratios may be useful alternative markers for estimating IR in various races [7].

In the current study, TG/HDL-C, TC/HDL-C, and TyG indices strongly correlated with IR as estimated by HOMA-IR, FG-IR, and QUICKI indices among Iranian women diagnosed with PCOS. An increase in these ratios positively correlated with HOMA-IR index and negatively correlated with the FG-IR and QUICKI indices even after adjusting for BMI and age. Although the IR group showed no dyslipidemia, TG/HDL-C, TC/HDL-C, and TyG ratios were significantly higher in the subjects with a HOMA-IR value of ≥2.63 (HOMA-IR-positive) compared to those with a HOMA-IR value of< 2.63 (HOMA-IR-negative). Besides, the IR group had a significantly higher TC/HDL-C ratio as measured by the FG-IR and QUICKI indices. A comprehensive analysis of the TG/HDL-C, TC/HDL-C, and TyG ratios using several IR indices suggested that these ratios might be more useful indicators of IR than the lipid profiles. Increased levels of TG, TC, and FBG and a decreased level of HDL-C are characteristic of IR and metabolic syndromes. Therefore, an elevation in these ratios is a strong preliminary predictor of IR and its associated complications, including reproductive disorders and metabolic abnormalities (glucose intolerance, T2DM, dyslipidemia, inflammation, oxidative stress, and CVD) which are more common in PCOS women [26]. Furthermore, these ratios have been viewed as practical methods in detecting IR in previous studies [29]. To the best of our knowledge, this study is the first comprehensive study investigating these relationships among Iranian women diagnosed with PCOS.

In this context, some studies have also reported the performance of these ratios in IR assessment. The positive correlation of TG/HDL-C and TC/HDL-C with the HOMA-IR index has been demonstrated among PCOS women in China [25] and diabetic patients in Thailand [30]. Also, several studies have advocated that TG/HDL-C ratio could be used as an indicator of IR in some populations, including the middle-aged and elderly populations in Taiwan [16], euthyroid normal-weight healthy adults in Peru [31], PCOS females in India [32] and non-diabetic subjects in China [29]. A large-scale cross-sectional analysis among Chinese adults also indicated that the TG/HDL-C ratio and TyG index are useful markers in estimating the IR and the TyG index is the best indicator for assessing IR risk [33]. It is important to highlight that, in spite of the clinical effectiveness of these ratios, inconsistencies have been identified with respect to ethnicity [31]. For example, researches conducted in the African-Americans populations have revealed that the TG/HDL-C ratio is not a reliable indicator for IR assessment [34–36].

Ghaffarzad et al. conducted a study on 36 Iranian infertile women with PCOS and reported a significant association between the TC/HDL-C, TG/HDL-C, and LDLC/HDL-C ratios with IR (as estimated by the HOMA-IR index) [7]. Hadaegh et al. conducted another study with 5201 non-diabetic Iranian subjects and found that the TG/HDL-C and TC/HDL-C ratios served as independent predictors of diabetes incidence during ≈6 years of follow-up [37]. Although these studies have demonstrated the efficiency of lipid ratios for IR prediction, there is little information on the lipid ratios and TyG index among PCOS women within the Iranian population. Moreover, most studies in this field have been done based on the HOMA-IR index, disregarding the FG-IR and QUICKI indices as two well-known indicators for IR. Given this, the present study aimed to assess the diagnostic ability of lipid profile, TG/HDL-C, TC/HDL-C, and TyG in classifying IR. ROC curve analysis was applied using the control cut-off values for the HOMA-IR and FG-IR indices (i.e., 2.63 and 8.25, respectively). The lipid profile did not have a sufficient predictability for IR. However, the AUC values of TG/HDL-C, TC/HDL-C, and TyG were considered to be acceptable for predicting the HOMA-IR and FG-IR indices. Thus, it seems that, consistent with prior evidence [7], these ratios are effective and beneficial diagnostic indicators for IR in the PCOS. In the current study, TC/HDL-C ratio based on FG-IR and TyG based on HOMA-IR had the highest AUC value. This finding is contrary to the results from a previous study showing that TG/HDL-C based on HOMA-IR index had the highest AUC value [7]. This discrepancy could be partially due to the larger sample size recruited in the present research. Although, the diagnostic accuracy of the TyG index has been described in some populations [38–40], the current study is the first to assess the diagnostic ability of TyG among the Iranian women with PCOS.

In this study, the optimal cut-off points of TG/HDL-C, TC/HDL-C, and TyG for predicting IR using the model based on the HOMA-IR levels were 2.8 (sensitivity of 60%, specificity of 56%), 4 (sensitivity of 60%, specificity of 57%), and 4.65 (sensitivity of 63%, specificity of 60%), respectively. The optimal cut-off value of TC/HDL-C using the model based on the FG-IR levels was 4.1 (sensitivity of 60%, specificity of 58%). In a previous study with a smaller sample size of Iranian women with PCOS, the best cut-off point of TG/HDL-C and TC/HDL-C levels were 3.19 )sensitivity of 63.6%, specificity of 84.4%) and 4.37 )sensitivity of 69.7%, specificity of 65.6%), respectively [7]. In another study on the young Korean women with PCOS, the cut-off value of the TG/HDL-C ratio to predict the IR (estimated by the HOMA-IR index) was reported as 2.5 (sensitivity of 61%, specificity of 82%) [41].

In the current study, homocysteine levels were significantly higher in the IS group only when the HOMA-IR index was used as an estimate of IR, showing an inverse relationship with HOMA-IR and a direct one with FG-IR and QUICKI. This came in accordance with a study by Jacobsen et al., where an unexpected inverse correlation was observed between IR and serum homocysteine in healthy subjects [42]. Fonseca et al. also reported that the IS subjects had higher levels of homocysteine [43]. Furthermore, it has been demonstrated that IR and serum homocysteine levels correlate with BMI and age [44]. In the present study, the two groups were similar in terms of BMI and age. It seems that IR is not the only factor in prompting hyperhomocysteinemia in PCOS women. For example, increased homocysteine levels may be a result of vitamin B12 and folic acid deficiency. Accordingly, increased serum homocysteine level might be independently related to PCOS.

## Study strengths and limitations

This study is the first comprehensive study investigating the different metabolic indices for estimating IR -as assessed using the HOMA-IR, FG-IR, and QUICKI indices- among PCOS women in Iran. Several limitations can be noted in this study. For example, the lack of direct IR evaluation and the fact that the patients were evaluated only precluded the minimization of within-subject biological variation in the measurement of biochemical parameters. Other limitations include the patients’ unclear status of vitamin B12 and folic acid, as well as the disproportionate sample size. Future studies using a proportionate sample size are suggested among IR positive and IR negative PCOS women.

## Conclusion

IR has a crucial role in the pathogenesis of the PCOS. Additionally, dyslipidemia is a common metabolic disorder among PCOS women. Our findings indicated that the lipid profile values were not useful indicators for IR assessment in the PCOS. Instead, the findings revealed that the elevated levels of TyG, TG/HDL-C, and TC/HDL-C indices were significantly associated with IR among the Iranian women diagnosed with PCOS. Thus, it can be concluded that these indices are valuable indicators to predict the IR, partly due to their analytical and financial ease-of-access in all clinical laboratories. The use of TG/HDL-C, TC/HDL-C, and TyG indices is recommended in the assessment of IR risk among Iranian women with PCOS. Further epidemiological research is advised.

## Supplementary Information

**Additional file 1: Figure S1.** The results of ROC curve analysis regarding the predictability of the lipid profiles in classifying the IR considering (A) HOMA-IR and (B) FG-IR in the patients with PCOS.

## Data Availability

All data generated or analyzed during this study are included in this published article and its supplementary information file.

## Abbreviations

BMI: Body Mass Index
CVD: Cardiovascular Disease
FBG: Fasting Blood Glucose
FGIR: Fasting Glucose to Insulin Ratio
FSH: Follicle-Stimulating Hormone
HDL-C: High-Density Lipoprotein Cholesterol
HOMA-IR: Homeostasis Model Assessment of IR
IR: Insulin Resistance
IS: Insulin Sensitive
LDL-C: Low-Density Lipoprotein Cholesterol
LH: Luteinizing Hormone
NAFLD: Non-Alcoholic Fatty Liver Disease
PCOM: Polycystic Ovarian Morphology
PCOS: Polycystic Ovary Syndrome
QUICKI: Quantitative Insulin Sensitivity Check Index
ROC: Receiver Operator Characteristic
T2DM: Type 2 Diabetes Mellitus
TC: Total Cholesterol
TC/HDL-C: Total Cholesterol to High-Density Lipoprotein Cholesterol
TG: Triglyceride
TG/HDL-C: Triglyceride to High-Density Lipoprotein Cholesterol
TyG: Triglyceride-Glucose
