# Supplementary material for: Evaluation of lipid ratios and triglyceride-glucose index as risk markers of insulin resistance in Iranian polycystic ovary syndrome women
Source: Lipids Health Dis. 2020 Nov 8;19:235. doi: 10.1186/s12944-020-01410-8 (PMC7648985; doi:10.1186/s12944-020-01410-8)
Supplement: Supplementary file 1 — Additional file 1: Figure S1. The results of ROC curve analysis regarding the predictability of the lipid profiles in classifying the IR considering (A) HOMA-IR and (B) FG-IR in the patients with PCOS. [file 12944_2020_1410_MOESM1_ESM.docx]

Additional file 1: Fig 1. The results of ROC curve analysis regarding the predictability of the lipid profiles in classifying the IR considering (A) HOMA-IR and (B) FG-IR in the patients with PCOS


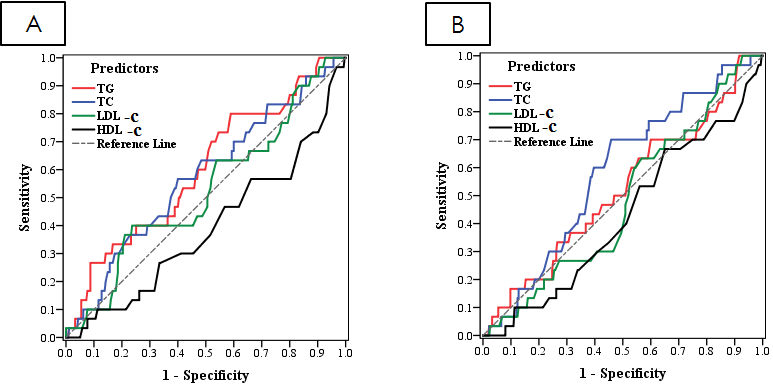


**Supplementary Fig 1.** The results of ROC curve analysis regarding the predictability of the lipid profiles in classifying the IR considering (A) HOMA-IR and (B) FG-IR in the patients with PCOS
